# A global database for modeling tumor-immune cell communication

**DOI:** 10.1038/s41597-023-02342-5

**Published:** 2023-07-12

**Authors:** Yunjin Xie, Weiwei Zhou, Jingyi Shi, Mengjia Xu, Zijing Lin, Donghao Li, Jianing Li, Shujun Cheng, Tingting Shao, Juan Xu

**Affiliations:** 1 College of Bioinformatics Science and Technology, Harbin Medical University, Harbin, 150081 China; 2 Endocrinology department, the first affiliated hospital of Harbin Medical University, Harbin, 150081 China; 3 State Key Laboratory of Molecular Oncology, Department of Etiology and Carcinogenesis, Cancer Institute and Hospital, Peking Union Medical College and Chinese Academy of Medical Sciences, Beijing, 100021 China

**Keywords:** Cancer microenvironment, Databases

## Abstract

Communications between tumor cells and surrounding immune cells help shape the tumor immunity continuum. Recent breakthroughs in high-throughput technologies as well as computational algorithms had reported many important tumor-immune cell (TIC) communications, which were scattered in thousands of published studies and impeded systematical characterization of the TIC communications across cancer. Here, a comprehensive database, TICCom, was developed to model TIC communications, containing 739 experimentally-validated or manually-curated interactions collected from more than 3,000 literatures as well as 4,537,709 predicted interactions inferred via six computational algorithms by reanalyzing 32 scRNA-seq datasets and bulk RNA-seq data across 25 cancer types. The communications between tumor cells and 14 types of immune cells were characterized, and the involved ligand-receptor interactions were further integrated. 14190 *human* and 3650 *mouse* integrated ligand-receptor interactions with supplemented corresponding function information were also stored in the TICCom database. Our database would serve as a valuable resource for investigating TIC communications.

## Background & Summary

Modulating the patient immune system with immunotherapy had revolutionized cancer therapy, and led to durable remissions across various cancer types^1,2^. The communications within or between the tumors and surrounding immune cells helped shape the tumor immunity continuum through chemokine-receptor signaling, and may contribute to different responses to immunotherapies^3–5^. Recent breakthroughs in cancer immunotherapy and decreasing costs of high-throughput technologies had sparked intensive research into tumor-immune cell (TIC) interactions. Single-cell RNA-seq (scRNA-seq) had been widely used to explore the cell composition of the tumor microenvironment in various cancer types, as well as the communication within or among these compositions^6–8^. Indeed, many studies had reported the important roles of TIC communications; however, this experimentally supported communication information was hidden in thousands of published studies^9–11^. These fragmented and even inconsistent publications were obstacles to characterizing TIC communications in the tumor microenvironment in both pan-cancer and tissue-specific contexts. Notably, no database was developed to collect these latest and experimentally supported TIC associations.

The communication between tumors and immune cells in the local tumor microenvironment started with the binding of a ligand to its receptor and the activation of specific cell signaling pathway^9,10,12^. Thus, collecting ligand-receptor interactions was fundamental to understanding TIC communication. For example, CellPhoneDB provided a resource of ligands, receptors, and their interactions, which took into account the subunit architecture of receptors^13^. In addition, many ligand-receptor interactions were collected by different prediction methods and resources of cell-cell communications, such as iTALK^14^, ICELLNET^15^, CellChat^16^. Thus, assembling these ligand-receptor interactions was an urgent task and would improve the prediction accuracy of cell communication.

To address this gap, we developed a comprehensive resource called TICCom to collect and integrate TIC communications. TICCom included not only communications supported by experiments and manual curation from the published literature, but also predicted results by several commonly used computational methods based on integrative ligand-receptor interactions and verified TIC interactions. Moreover, the TIC communications were further classified into three types based on the interaction model. We further manually labeled all the ligand-receptor pairs functionally, and much more other information was also provided, including expression of TIC communications across 33 major cancer types, their potential as prognostic markers and TF/miRNA regulation. Several flexible tools were developed to aid retrieval and analysis. TICCom would serve as a valuable resource for investigating communications between tumors and immune cells and greatly extend our understanding of cancer immunotherapy.

## Methods

### Collection of TIC communications from literature

**Fig. 1:**
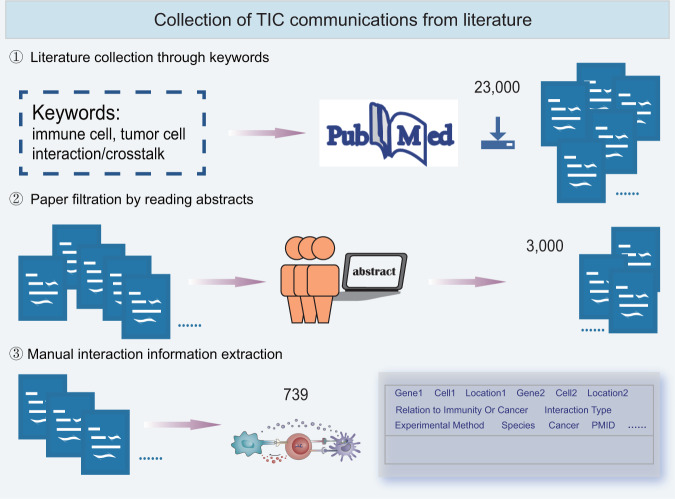
Collection of TIC communications from literature. Firstly, ~23,000 articles were downloaded from PubMed and retrieved using keywords. Secondly, three researchers carefully read abstracts, and 3000 articles were retained. Finally, detailed information on tumor-immune cell communication was extracted.

Firstly, an extensive literature query of the PubMed database was performed using a list of keywords, such as ‘immune cell’, ‘tumor cell’, ‘crosstalk’ and ‘interaction’. ~23,000 references were retrieved, the titles and abstracts of which were downloaded. Secondly, we filtered the papers related to tumor-immune interactions by reading the abstracts. Lastly, more than 3,000 literatures remained, and the TIC interaction information was manually extracted from these literatures, including interaction gene pairs, functions, subcellular localizations, experimental methods, descriptions of interactions, titles and PMIDs of literatures, and other details (Fig. 1).

### Integration of ligand-receptor interactions data

14,190 *human* ligand-receptor (LR) pairs were collected from seven studies^7,14–19^, while 3,650 *mouse* LR pairs were collected from two studies^19,20^. Genes were represented by Ensembl gene IDs. Considering the immunogenicity of LR interactions, we classified LR pairs into 10 groups: notch signaling, antigen binding, neuropeptide, hormone, growth factor, interferon, interleukin, tumor necrosis factor, chemokine, and cytokine by simultaneously annotating ligands and receptors to relevant GO terms obtained from MSigDB^21^. For example, if a ligand and its coupled receptor were annotated to cytokine-related GO term groups, this pair was assigned to the cytokine group. Unsuccessfully assigned LR pairs were finally appointed to the ‘other’ group. In order to facilitate the further understanding of the confidence of LR interactions, these integrated interactions were grouped into two subclasses based on their identification methods in previous studies. The interactions were labeled ‘manually curated’ if they were supported by experimental data or manual annotation from the literature in at least one dataset, and others were grouped into the predicted subclass.

### Cancer transcriptome datasets

Bulk RNA-seq data were collected and unified from The Cancer Genome Atlas (TCGA, https://portal.gdc.cancer.gov/), the International Cancer Genome Consortium (ICGC, https://dcc.icgc.org/)^22^, and the EMBL-EBI Expression Atlas (https://www.ebi.ac.uk/gxa/home)^23^, including 12,914 samples of 25 cancer types. The cancer transcriptome datasets are shown in Supplementary Table 1. The count matrix of genes was quantified as fragments per kilobase per million reads mapped (FPKM). Genes whose expression was zero in more than 30% of samples were excluded. The expression was log2(FPKM + 0.05) normalized. In addition, 32 single-cell RNA-seq datasets from 13 cancer types having both tumor cells and immune cells were retrieved from both the NCBI Gene Expression Omnibus (GEO)^24,25^ and the TISCH database^26^. The scRNA-seq datasets are displayed in Table 1. As previous works^27–29^, we predicted cell-cell interactions separately based on each scRNA-seq dataset containing more than 500 cells or bulk RNA-seq dataset with at least six samples. Cancer categories were carefully unified and listed in Supplementary Table 2.

**Table 1:** scRNA-seq data obtained from GEO and TISCH.

Cancer Type	Cancer Type Detailed	Source	Source2
Bladder Cancer	Bladder Cancer	GSE145137^37^	GEO^24,25^
Leukemia	Acute Lymphocytic Leukemia	GSE132509^38^	TISCH^26^
	Acute Erythroid Leukemia	GSE142213^39^	TISCH
	Acute Myeloid Leukemia	GSE116256^40^	GEO
Breast Cancer	Breast Cancer	GSE143423^41^	TISCH
		GSE75688^42^	GEO
		SRP114962^43^	TISCH
Brain Cancer	Glioma	GSE102130^44^	TISCH
		GSE103224^45^	TISCH
		GSE138794^46^	TISCH
		GSE139448^47^	TISCH
		GSE141982^48^	GEO
		GSE70630^49^	TISCH
		GSE84465^50^	GEO
		GSE89567^51^	TISCH
Head and Neck Cancer	Head and Neck Squamous Cell Carcinoma	GSE103322^52^	GEO
Colorectal Cancer	Colorectal Cancer	GSE146771^53^	GEO
Liver Cancer	Liver Cancer	GSE125449^54^	GEO
Lung Cancer	Lung Adenocarcinoma	GSE131907^55^	GEO
	Non Small Cell Lung Carcinoma	EMTAB6149^56^	TISCH
		GSE117570^57^	TISCH
		GSE127465^58^	TISCH
		GSE143423^41^	TISCH
Neuroendocrine Cancer	Neuroendocrine Cancer	GSE140312^59^	TISCH
Ovarian Cancer	Ovarian Cancer	GSE118828^60^	TISCH
Pancreatic Cancer	Pancreatic Adenocarcinoma	CRA001160^61^	TISCH
		GSE111672^62^	TISCH
Skin Cancer	Skin Cutaneous Melanoma	GSE115978^63^	GEO
		GSE72056^64^	GEO
	Basal Cell Carcinoma	GSE123813^65^	GEO
	Merkel Cell Carcinoma	GSE117988^66^	GEO
	Uveal Melanoma	GSE139829^67^	TISCH
Gastric Cancer	Early Gastric Cancer	GSE134520^68^	GEO

### Computationally predicting TIC communications

TICCom provided inferred cell-cell communication based on 32 scRNA-seq datasets of 13 cancer types through five popular computational methods according to their corresponding standardized processes, including iTALK-top^14^, iTALK-DEG^14^, CellTalker^30^, ICELLNET^15^, and NicheNet^17^. The inferred TIC communication was characterized by providing detailed information, including gene names, functions, and labels (evidence) for prediction or manual curation, as well as computational methods and cancer types.

Interaction strengths of TIC communication based on bulk RNA-seq data and the integrated LR interactions were estimated by TItalk provided by TICCom. All genes were ranked in descending order by their expression for each sample. Interaction strength of a pair of interacting genes ISg for a sample n was defined as follows:

$${{\rm{ISg}}}_{{\rm{n}}}=({{\rm{rank}}}_{{\rm{n}}1}+{{\rm{rank}}}_{{\rm{n}}2})\times (10-{\rm{abs}}({{\rm{rank}}}_{{\rm{n}}1}-{{\rm{rank}}}_{{\rm{n}}2}))$$

where rank_n1_ and rank_n2_ were the positions of two interacting genes in the sorted vectors according to their expression in sample_n_ respectively. The statistical significance was calculated as the probability of observing a lower interaction strength than the true one through 1000 random samplings. The datasets consisted of predicted interaction strengths and p values of gene interactions occurring between tumor and immune cells across cancer types.

### miRNA-target and TF-gene interactions

17,723 miRNA-target interactions were downloaded from starBase^31^, and 29,251 TF-gene interactions were downloaded from TRRUST^32^, HTRIdb^33^, and ORTI^34^.

## Data Records

These datasets can be obtained from Figshare^35^ and from the download page of TICCom (http://bio-bigdata.hrbmu.edu.cn/TICCom/). The R codes used to generate datasets in TICCom were shared on Github (https://github.com/yunjinxie/TICCom-dataset).

There were five CSV files in the database. All the deposited data was processed, and all the sources were openly available. The CSV table ‘Experimentally verified TIC communication’ contained detailed information on experimentally-verified TIC communications, including gene symbols, cell types, interaction types, species, experiments, interaction comments, and original reference information. Integrated ligand-receptor interactions were displayed in the CSV table ‘Integrated ligand-receptor interactions’, including gene symbols, functions, sources of LR pairs, and evidence. Predicted TIC communications based on bulk RNA-seq were stored in the CSV tables ‘Predicted TIC communication based on experimentally verified TICs and bulk RNA-seq’ and ‘Predicted TIC communication based on ligand-receptor interactions and bulk RNA-seq’. Information included gene symbols, cell types, cancer types, interaction strengths, and p values in the former dataset. However, in the latter dataset, ligands, receptors, functions, evidence, cancer types, interaction strengths, and p values were recorded. The CSV table ‘Predicted TIC communication based on scRNA-seq’ contained detailed information about predicted TIC interactions inferred by five algorithms using 32 scRNA-seq datasets and the integrated ligand-receptor interactions. Ligands, receptors, cell types, prediction methods, evidence, datasets, cancer types, and cancer subtypes were included in this dataset. In Supplementary Table 3, the columns of five CSV files are listed.

## Technical Validation

**Fig. 2:**
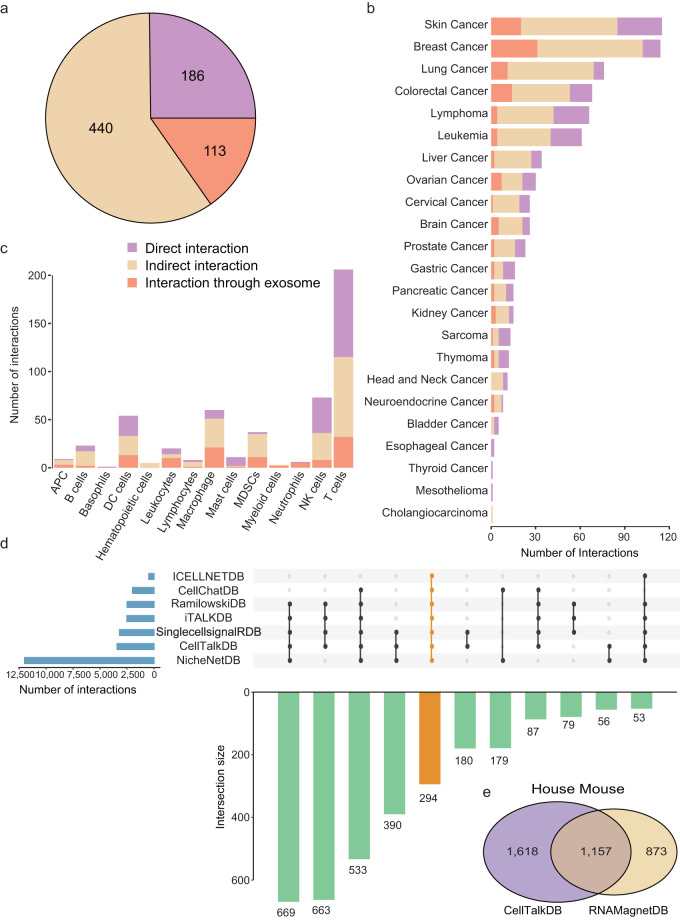
The resources in TICCom. (**a**) The number of experimentally-verified tumor-immune cell interactions of different interaction types. (**b**) The number of verified tumor-immune cell interactions occurred in 23 cancer types. (**c**) The number of verified tumor-immune cell interactions occurred in 14 immune cells. (**d**) The total number of ligand-receptor interactions across seven *human* datasets was displayed in a bar plot on the left. The seven *human* datasets were represented by dots in the corresponding rows of the dot matrix, which also showed the intersection set of the datasets in its column. The intersection set size was depicted by the bar plot at the bottom. (**e**) The Venn plot showed the number of ligand-receptor interactions shared by the two *mouse* datasets, CellTalkDB and RNAMagnetDB. The left and right circles indicated the number of ligand-receptor interactions in CellTalkDB and RNAMagnetDB, respectively.

**Fig. 3:**
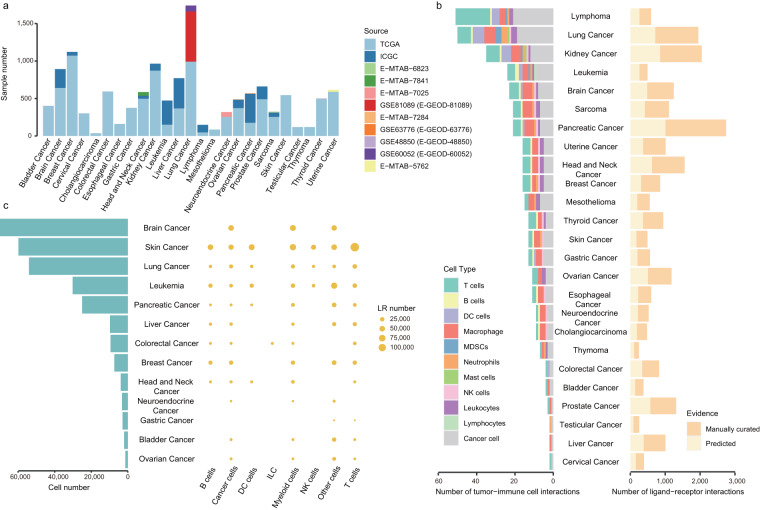
The predicted interactions stored in TICCom. (**a**) The number of samples in each cancer type from different bulk RNA-seq resources. The datasets with serial numbers containing MTAB or GSE were obtained from the EMBL-EBI Expression Atlas. (**b**) The number of predicted verified tumor-immune cell direct interactions and that of predicted ligand-receptor interactions in bulk RNA-seq data from 25 cancer types. The significant p values of TCGA and ICGC cancer were set at 0.05; however, the significant p value of EMBL was set at 0.1 because of the smaller number of samples. (**c**) The bubble plot showed the number of predicted ligand-receptor interactions that occurred in each cancer and each immune cell inferred by five algorithms based on 32 scRNA-seq datasets from 13 cancer types and the integrated ligand-receptor interactions. The bar plot showed the number of cells in each cancer. The size of the bubble indicated the number of predicted ligand-receptor interactions.

In order to validate the accuracy of experimentally supported TIC communications, the process of data extraction was performed independently by Y.X., J.S., and M.X. and subsequently cross-checked. The resolution of any disagreements regarding data extraction was based on consensus. Information retrieval was done manually. The dataset included 739 experimentally-verified TIC interactions from *human* and *mouse*, covering 26 years of experiments from Jan. 1993 to Jul. 2019. We proofread and validated the TIC communication by pulling low-throughput experiments from articles such as immunoprecipitation assays, qPCR, ELISPOT assays and Western blot assays. These interactions were divided into three categories: (1) 186 direct interactions, which meant these interactions directly occurred between tumor cells and immune cells; (2) 113 secretory interactions, which meant molecules derived from tumor cells or immune cells bound to corresponding receptors influencing TIC communication; and (3) 440 indirect interactions, which meant these interactions occurred within tumor cells or immune cells and were essential to tumor immunity (Fig. 2a). We carefully unified the names of cancer categories and immune cells based on the clinician’s suggestions. Cancer categories are listed in Supplementary Table 2. These interactions involved 14 immune cells and 57 cancer subtypes of 23 cancer types (Fig. 2b,c, Supplementary Table 2). The number of TIC interactions varied across different tumors. This may be due to that the majority of experimentally-verified TIC interactions originate from these cancers, such as skin cancer, breast cancer, and lung cancer, in which tumor immunity has been a research hotspot, and cancer cell-immune cell crosstalk in other cancers may not have been explored in depth. In addition, the number of LR interactions varied across these seven *human* LR interaction datasets. Only 294 LR interactions were shared by seven *human* datasets (Fig. 2d). There were 1,157 common LR interactions in two *mouse* LR interaction datasets, accounting for 42% and 57% of the total, respectively (Fig. 2e). In order to obtain more comprehensive and precise resources on LR interactions, 14,190 *human* LR interactions and 3,650 *mouse* LR interactions were integrated from seven *human* datasets and two *mouse* datasets, respectively. We unified the gene symbols and Ensembl gene IDs of all the involved genes. The functions of these LR interactions were annotated by hand. In order to strengthen the credibility of LR interactions, these integrated interactions were grouped into the manually curated subclass or the predicted subclass based on their identification methods in previous studies. To guarantee the precision of interaction strength predicted by the Interaction Intensity module and TItalk provided by TICCom, we designed a p value that determined whether the real interaction strength was larger than the random one. The interaction strength was inferred based on bulk RNA-seq data from 25 cancer types (Fig. 3a,b, Supplementary Table 1). In addition, to ensure the accuracy of the predicted cell-cell crosstalk based on 32 scRNA-seq datasets from 13 cancer types (Fig. 3c, Table 1), we combined the results inferred from five algorithms in the Prediction module and used the integrated LR interactions as reference interactions. Before uploading these five datasets into TICCom, we re-checked the metadata of the MySQL database.

**Fig. 4:**
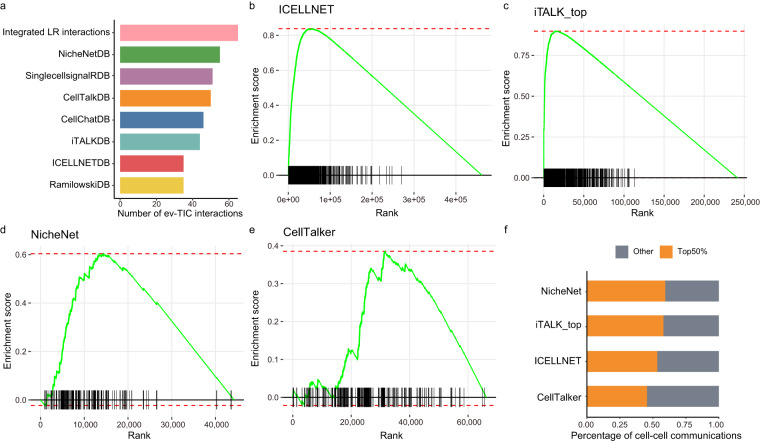
The improvement of TICCom for prediction accuracy. (**a**) The number of experimentally-verified TIC (ev-TIC) communications in seven *human* resources and the integrated LR interactions. (**b-e**) The results of gene set enrichment analysis. To further illustrate the improvement of TICCom in prediction accuracy, we applied different algorithms, including ICELLNET, iTALK-top, NicheNet, and CellTalker, to infer cell-cell communication based on the integrated ligand-receptor interactions and iTALKDB LR interaction dataset in basal cell carcinoma (GSE123813^65^). The prediction result integrated from predicted cell-cell interactions inferred by these algorithms was used as the gene set. The prediction result of a single method was used as the ordered gene list, ranked by the communication score. (**f**) The percentage of top 50% of the cell-cell communication inferred from a single method occupied in the integrated result.

To improve the prediction accuracy of cell communication, two different aspects were considered by TICCom: the integration of ligand-receptor (LR) pair datasets and the integration of cell-cell interactions predicted based on different algorithms. We first assessed the contribution of integrating LR interaction datasets to prediction accuracy compared to a single LR resource. The result showed that the integrated data contained more experimentally-verified TIC interactions than single resources (Fig. 4a). On the other hand, we evaluated whether the integrated predicted cell-cell interactions tended to be optimized preferentially by each individual algorithm. We found that integrated predicted cell-cell interactions had higher communication scores in each algorithm by Gene Set Enrichment Analysis (GSEA) (Fig. 4b–e). Additionally, more than half of integrated predicted cell-cell communications accounted for the top 50% of results predicted by individual algorithms (Fig. 4f). The results indicated that integrating both LR interaction datasets and cell-cell interactions predicted by different algorithms could improve prediction accuracy at different levels.

## Supplementary information

Supplemental Table 1

Supplemental Table 2

Supplemental Table 3

## Data Availability

The R codes used to generate datasets in TICCom were shared on Github^36^ (https://github.com/yunjinxie/TICCom-dataset) with the identifier (10.5281/zenodo.8060109). All software tools used in this study are freely available.
